# In non-transplant patients with multiple myeloma, the pre-treatment level of clonotypic cells predicts event-free survival

**DOI:** 10.1186/1476-4598-11-78

**Published:** 2012-10-19

**Authors:** Kyle J Thulien, Andrew R Belch, Tony Reiman, Linda M Pilarski

**Affiliations:** 1 Department of Oncology, University of Alberta and Cross Cancer Institute, 11560 University Avenue, Edmonton, AB, T6G1Z2, Canada

**Keywords:** Multiple myeloma, Clonotypic signature, Quantitative analysis, Clinical correlates, Cancer stem cell, Autologous transplant, Tumor burden

## Abstract

**Background:**

In multiple myeloma (MM), the immunoglobulin heavy chain VDJ gene rearrangement is a unique clonotypic signature that identifies all members of the myeloma clone independent of morphology or phenotype. Each clonotypic MM cell has only one genomic copy of the rearranged IgH VDJ.

**Methods:**

Pre-treatment bone marrow aspirates from myeloma patients at diagnosis or in relapse were evaluated for the number of clonotypic cells using real time quantitative PCR (RPCR). RPCR measured the level of clonal cells, termed VDJ%, in 139 diagnosis and relapse BM aspirates from MM patients.

**Results:**

Patients with a VDJ% below the median had a significantly longer event free survival (EFS) then those with a VDJ% higher than the median (p=0.0077, HR=0.57). Further, although the VDJ% from non-transplant patients predicted EFS (p=0.0093), VDJ% failed to predict outcome after autologous stem cell transplant (p=0.53).

**Conclusions:**

Our results suggest that for non-transplant patients, the tumor burden before treatment, perhaps reflecting cancer stem cell progeny/output, is an indirect measure that may indicate the number of MM cancer stem cells and hence event free survival.

## Introduction

Multiple myeloma (MM) is a clonal B-cell malignancy with clinical identification of plasma cells (PCs) that accumulate in the bone marrow (BM). Newer treatments continue to increase the incidence of remission and decrease side effects, but relapse invariably occurs. Although the MM clone is known to be heterogeneous, including earlier stage B cells [1-6] and putative cancer stem cells [4-7] as well as PC, current diagnostic and monitoring criteria are based mainly on BM plasmacytosis (BMPC) and serum M-protein levels (Mpr) as measured in the clinical laboratory. Although measures of BMPC morphology and Mpr are clinically informative [8-12], they are not among the parameters recommended for risk stratification [13,14]. The IgH VDJ gene rearrangement that characterizes the MM clone in each patient, termed clonotypic, is a molecular signature that identifies all malignant components regardless of differentiation stage, phenotype, morphology or other molecular markers [2,15]. Even though abnormal circulating or BM PC phenotypes [12,16-19] provide informative prognostic measures, particularly for autologous stem cell transplantation, it would be of value to confirm that the malignant cells themselves express these markers, as well as providing additional predictive value [20-25]. Molecular analysis may also be important for evaluating risk stratification in patients receiving novel therapies [26-28].

Because each malignant cell harbors only one copy of the clonotypic IgH VDJ, using quantitative PCR the malignant clone can be enumerated in genomic DNA from a heterogeneous population of cells. Here we used SYBR Green real-time quantitative PCR (RPCR) with patient specific CDR2/CDR3 primers to measure the fraction of clonal cells among total BM cells, termed VDJ%, in 139 diagnosis and relapse BM aspirates from MM patients. The results presented below suggest that the VDJ% from the diagnosis and relapse BM prior to initiating a given treatment is an independent measure of the remission length in non-transplant patients, unlike BMPC or Mpr. While standard clinical tests failed to predict remission duration, the percent of clonal cells as measured in BM aspirates with RPCR stratified patients into high risk and low risk categories. The VDJ% enumerates all cells containing the clonal sequence and thus may include relevant cell types not included among BMPC or contributing to Mpr levels. Pre-treatment VDJ% was independent of disease response and drug choice suggesting that absent autologous stem cell transplant, it may be the specific biology of each tumor that determines remission length and not the specific treatment.

## Results

Although it has been well documented that clinically-measured levels of residual disease during remissions of hematological cancers can be significantly associated with event free survival, there has been little documentation of the impact on outcome of total tumor burden prior to treatment. This study utilizes patient-specific RPCR to molecularly estimate the total tumor burden, as defined by the IgH VDJ molecular signature, in the BM of MM patients. Patient-specific RPCR quantifies all cell types harboring the clonotypic signature, whether or not they are clinically detectable.

### Sensitivity of the RPCR assay

**Figure 1:**
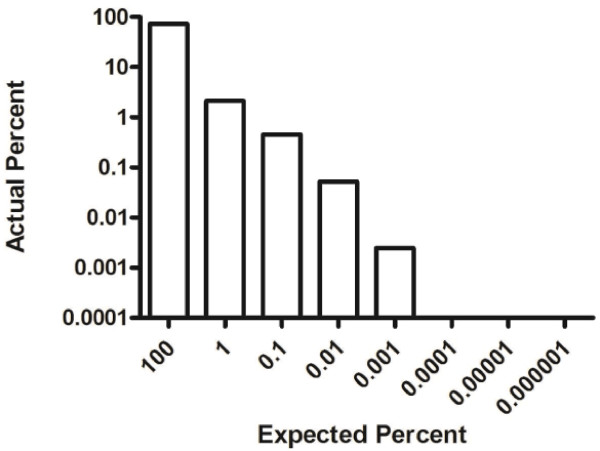
**Sensitivity of patient-specific RPCR.** The bars represent the mean of 3 replicate PCR reactions to determine the VDJ% for each dilution noted by comparing the VDJ PCR molecule count plotted from the VDJ control curve to the β2m molecule count using half the DNA. The absence of bars at the 0.0001%, 0.00001% and 0.000001% levels indicate the absence of amplification.

To determine the lowest level of detection, DNA from a MM PC cell line (LP-1) with a known clonotypic IgH VDJ rearrangement was diluted in a constant amount of normal PBMC DNA to simulate patient samples with different clonal percentages. The average molecule count for each dilution, determined using a standard curve for the VDJ and β2m PCRs, was used to calculate the percent of clonal cells (Figure 1). The lowest dilution with detectable VDJ signal was 0.001%. Thus, patient samples that fell below this threshold value were arbitrarily assigned a value of 0.001% for inclusion in the analysis.

A limiting dilution assay was used to confirm the RPCR results from a BM sample. The VDJ% was calculated using the cell equivalents added and the PCR results for each dilution. For the same BM sample, the clonal percent estimate was 7.97% based on limiting dilution and 7.93% based on RPCR.

### Pre-treatment VDJ% correlates with EFS

**Figure 2:**
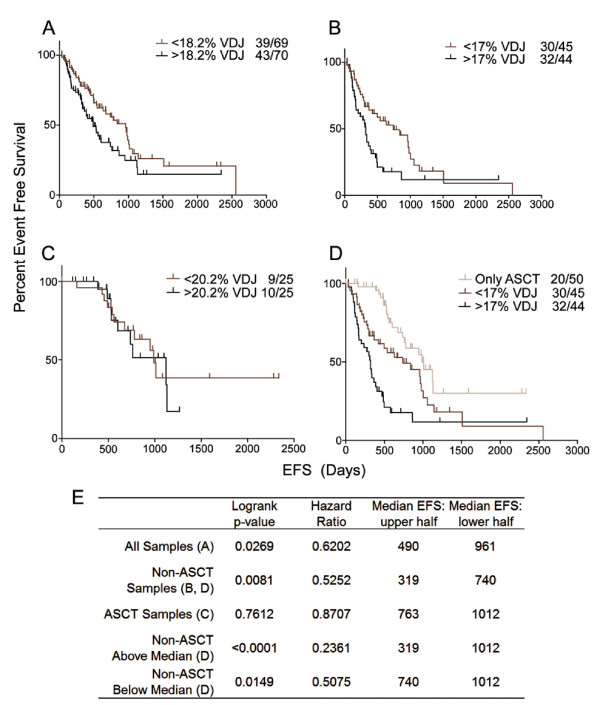
**Kaplan-Meier analysis of pre-treatment BM VDJ% and EFS.** The VDJ%, dichotomized by the median, shows a significant correlation with EFS (A) - those who fall below the median (light line) have longer EFSs than those who fall above the median (dark line). Statistical determination of the optimal cut point for dichotomizing into two groups gave a value nearly identical to the median (18.6%). There were 39 relapses out of 69 patient samples below the median and 43 of 70 above the median. When patient samples were stratified to exclude those receiving an ASCT (the group of non-transplant patients), the significance remains (**B**). When the remaining patients who did receive an ASCT were analyzed, the significant correlation was lost (**C**). **D** represents the Kaplan-Meier analyses for three significantly different EFS categories: lightest line=all ASCT treated patients, medium grey line= non-transplant patients below the VDJ% median, and the dark line= non-transplant patients above the VDJ% median, with respectively 20 relapses out of 50 samples, 30 out of 45 and 32 out of 44. **E** summarizes the logrank p-value, hazard ratio and median survivals for the above plots.

One hundred and thirty nine BM aspirate samples from MM patients were collected just before treatment, 89 of which were from previously untreated MMs and 50 were relapsed/refractory (RR) MMs starting a new line of therapy (Table 1). BM aspirates should be understood as sampling a geographically distinct location, a caveat that applies to all aspirate-based studies; the generally held but unproven working assumption is that the aspirate is representative of the BM as a whole. Comparison with standard curves gave a molecule count for VDJ targets and β2m targets. A percent of clonal cells in BMMC, termed VDJ% was then determined. Based on the VDJ%, the patient cohort was dichotomized into two groups: above or below the median of 18.2%. Event free survival (EFS), was significantly different between them, as shown by Kaplan Meier analysis (p=0.0269). Those patient samples with a VDJ% falling below the median had a longer EFS than those above the median (Figure 2A). The hazard ratio (HR) for patients below the median VDJ% compared to patients above the median VDJ% is 0.6202. The median EFS time for above and below the median VDJ% was 490 days and 961 days, respectively.

**Table 1:** Clinical characteristics: the high VDJ% group includes more patients with ASCT than with conventional chemotherapy

	**Untreated/ Chemo**	**RR/Chemo**	**Untreated/ASCT**	**RR/ASCT**
High VDJ%	16	24	28	1
Low VDJ%	24	25	21	0
Total	40	49	49	1

VDJ% in patients whose BM aspirates were taken at diagnosis or relapse and who ultimately received either conventional chemotherapy or ASCT.

The VDJ% prior to therapy was not associated with overall survival (not shown). A significant association was found between pre-treatment VDJ% and β2m, LDH, hemoglobin or creatinine, but not with any other factors noted in Table 2.

**Table 2:** Clinical characteristics of patients with high or low VDJ%

		**High VDJ%**	**High VDJ%**	**Low VDJ%**	**Low VDJ%**	**High VDJ%**	**Low VDJ%**	**Remission Samples**
		**Unt/Ch**	**RR/Ch**	**Unt/Ch**	**RR/Ch**	**ASCT**	**ASCT**	
Age	≥65	11/16	12/24	20/24	13/25	5/29	6/21	11/33
Albumin	>30	13/15	23/24	20/24	24/24	26/27	19/21	27/28
β2M	≥3	12/14	16/21	16/19	14/22	18/24	9/18	18/26
Calcium	≥2.5	1/14	8/23	5/24	5/24	7/26	6/21	11/29
Creatinine	≥177	3/14	5/24	6/24	0/24	7/28	1/21	4/29
Hemoglobin	>100	8/16	13/24	18/24	20/24	15/29	14/20	23/30
LDH	>618	6/14	2/24	2/22	2/23	6/25	1/21	3/28
t(4;14)	Positive	0/12	3/15	3/18	2/19	2/20	2/15	5/29
Stage	III	7/14	10/21	9/19	8/22	8/23	5/18	10/25

Unt=Untreated, RR = relapse/refractory, Ch = only conventional chemotherapy.

Sub-dividing the patient cohort to include only non- autologous stem cell transplant (ASCT) patients showed a significant association between VDJ% and EFS in a Kaplan Meier analysis (p=0.0081, HR=0.5252) (Figure 2B). In contrast, the VDJ% from the subset of patients who received an ASCT as part of their treatment regimen failed to show a significant association with EFS by Kaplan Meier analysis (p=0.5306, HR=0.7569) (not shown), despite the fact that a large proportion of patients who received ASCT fell into the high VDJ% group (Table 1). Significantly different EFS categories were observed between ASCT patients (1012 days), non-ASCT treated patients with a VDJ% below median (740 days) and non-ASCT treated patients with a VDJ% above the median (319 days) (Figure 2D, E). The prolonged survival for ASCT patients occurred even though a large proportion had a high VDJ% (Table 1).

In a Cox multivariate analysis adjusted for International Staging System (ISS), patients with a VDJ% higher than the median have a shorter EFS than do patients with a low VDJ% (p = .002 for non-ASCT and a trend for ASCT patients p=.11). In a Cox regression univariate model of the combined patient groups adjusted for ISS, patients with high VDJ% also have shorter EFS (p=.005).

### VDJ% but not BMPC significantly associates with EFS

**Figure 3:**
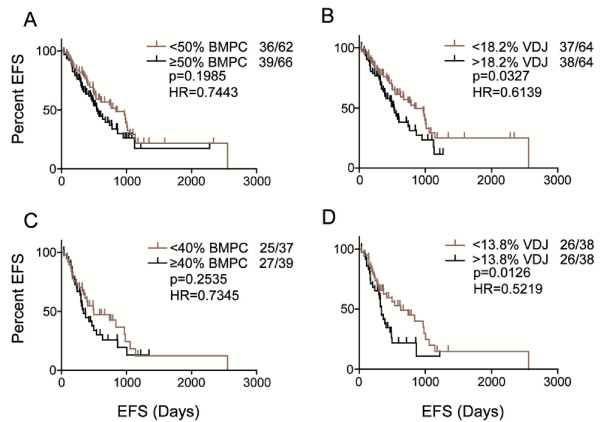
**Kaplan Meier Curves of BMPC or Matched VDJ% to EFS.** The median BMPC percentage dichotomized the group but did not indicate significantly different EFSs (A). The same patient cohort, categorized by VDJ% above and below the median, significantly correlates with EFS (**B**). If only non-transplant patient samples were included, the EFS compared to the BMPC remains insignificant (**C**) and the EFS compared to VDJ% remains significant (**D**).

Part of the clinical evaluation of MM is to determine the % PC in the BM aspirate, termed BM plasmacytosis (BMPC), a measure not thought to be associated with outcome. RPCR utilized DNA from pre-treatment BM samples for which BMPC values were available from the routine clinical analysis of BM aspirates. For the cohort analyzed here in a Kaplan Meier analysis where the median dichotomized the group, the BMPC failed to show a significant association with EFS (Figure 3A). Furthermore, no significant correlations were seen with any clinical outcome when monoclonal protein levels (Mpr) were evaluated (not shown). In contrast, when the VDJ% dichotomized the full cohort for a Kaplan Meier analysis, a significant correlation was observed. If only non-ASCT patients were included in the Kaplan-Meier analysis of BMPC and EFS, there continued to be no significant association (Figure 3C). Again in contrast, for VDJ%, a significant correlation with EFS was seen for the non-ASCT cohort (Figures 3B and D).

For a subset of 47 patients, we evaluated the relationship between %PC (as measured by flow cytometry) and VDJ% for the same sample of purified BMMC (data not shown). Given that the majority of clonotypic cells in untreated or relapsed/refractory MM BMMC are likely to be PC, the expected significant correlation was observed between %PC and VDJ% (p<.0001). This however does not allow us to determine the extent to which non-PC contribute to the VDJ%, as implied by the fact that VDJ% but not BMPC predicts EFS (Figure 3).

### The VDJ% from remission BMs may not associate with outcome

Little research has addressed the VDJ% for patients in remission after treatment for relapsed MM. In a preliminary analysis, for a small set of 32 relapsed patients who were subsequently treated and later achieved remission, the tumor burden in remission BM samples (post-treatment) was quantified using patient-specific RPCR. The samples were from different patients with different treatment courses, including frontline VAD/ASCT and bortezomib/ASCT (20/32 patients), or relapse samples treated with dexamethasone, bortezomib or lenalidomide (12/32 patients). Some remission samples did not have detectable VDJ%: these were arbitrarily assigned a value of 0.001%.

In a Kaplan Meier analysis, the remission VDJ% did not show a significant correlation with EFS (p=0.6934, HR=0.8026). Of the 25 patients with matched pre-treatment and remission samples, the relative reduction of VDJ% was calculated. This ‘relative reduction’ was not associated with EFS in a Kaplan-Meier analysis (p=0.4620, HR=0.6041).

As the remission BM samples were taken at varying times in the treatment course, time to progression (TTP) provides an alternate measure of outcome. TTP is calculated as the number of days between aspirating the remission BM and the time of clinical relapse. Neither the remission VDJ% or the relative reduction were significantly associated with TTP (p=0.4407 and p=0.5430, respectively) (data not shown).

**Figure 4:**
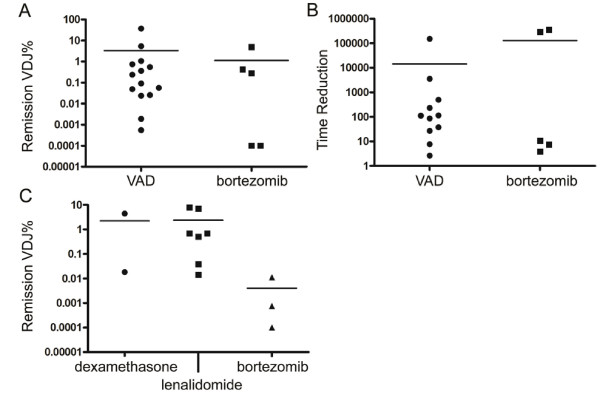
**Remission VDJ% and relative reduction of remission BM samples.** The post treatment VDJ% from a front-line trial of relapsed MM patients, comparing standard VAD to bortezomib just prior to ASCT (A). The times reduction of the VDJ% in the same samples are plotted in **B**. In relapsed patients, the VDJ% is noted in **C** after having been treated with dexamethasone, lenalidomide or bortezomib. The line through each column represents the mean. Dex=dexamethasone.

The remission VDJ% values and relative reduction were compared for VAD and bortezomib as frontline therapies (Figure 4A and B), and for dexamethasone, bortezomib or lenalidomide (Figure 4C). The number of patients is low, but no significant differences were found among these groups.

## Discussion

This study demonstrates that pre-treatment BM VDJ% has an inverse correlation with event free survival. The percent of clonal cells measured by RPCR in aspirated pre-treatment BM cells significantly correlated with EFS in a Kaplan-Meier analysis. Patients were treated with a variety of therapies for front-line and relapsed/refractory MM. VDJ% in non-transplant patients receiving chemotherapy or novel therapies (e.g. lenalidomide or bortezomib) correlated inversely with EFS, but VDJ% was not predictive for patients receiving an ASCT. Subsequently, three significantly different remission durations were identified. Patients receiving an ASCT had the longest remission duration. Patients not eligible for an ACST and having a VDJ% below the median (17% in this data set) had a longer remission duration than did those having a VDJ% above the median.

Others have shown that patients receiving high dose cytoreduction with auto- or allo-graft rescue are more likely to achieve molecular complete remissions and a subset of these patients have improved progression-free survival [29,30]. It appears that the benefits of complete remissions may occur mainly in the context of intensive therapy and stem cell rescue [11,21,31,32]. Consistent with these observations, the loss of correlation between pre-treatment VDJ% and outcome, compared to the non-transplant group, may further highlight the efficacy of high dose therapy followed by auto- or allo- stem cell transplant. It appears that an ASCT overcomes the contribution to outcome of pre-treatment tumor burden, even when that burden is high. Others have reported the pre-treatment levels of clonal cells [20,25,33] but did not evaluate associations between tumor burden and outcome. Most studies evaluated cohorts of patients who had received ASCT. Consistent with this, our work shows an absence of prognostic significance for the pre-treatment VDJ% in MM patients who ultimately received an ASCT.

It is important to note that most studies identifying EFS prognostic indicators, including serum β2m and albumin levels [34,35], chromosome 13 monosomy [36,37], and t(4;14) translocations [38], have evaluated samples from previously untreated patients. However, patients can only be ‘previously untreated’ once, and for the purposes of defining relapse risks for each cycle of therapy, the influence of these factors is less defined. Our study considers all the treatment cycles. VDJ% is predictive of EFS in untreated and relapsed MM, with the caveat that this correlation does not hold for patients who were ultimately treated with ASCT. As part of clinical testing, VDJ% may indicate to a treating clinician the current likelihood of early relapse.

In this study, the VDJ% from remission/post treatment BM samples could not predict outcome, albeit with only small numbers of patients. This is in contrast to previously published work, also on relatively small sample sets, showing that the frequency of clonal cells does correlate with EFS [29,32,39]. However, the small cohorts in previous studies were from uniformly treated patients, often collected at similar time points. In contrast, the remission BM samples used in this study were from patients treated with a variety of therapeutic regimens, although the majority (20/32) were in remission after relapsing from intensive therapy and ASCT. Samples were also collected from patients receiving lenalidomide, bortezomib, or dexamethasone for relapsed disease (12/32), taken at varying time points during remission. As no significant correlation was observed between the remission VDJ% or relative reduction in VDJ% after treatment and EFS from these unequal time points, the TTP was used to standardize the time points of BM sampling. Since the remission samples studied here were obtained from heterogeneous patient populations, the results may be broadly applicable. Analysis of a much larger cohort will be needed to determine whether or not these are equally effective treatments for reducing the disease burden as measured molecularly.

Although still very speculative, if the conclusion is correct that the remission BM VDJ% lacks a correlation with outcome, this may provide a new perspective on myeloma growth. The VDJ% enumerates all cells containing the IgH VDJ clonal sequence, regardless of their morphology or differentiation stage. In pre-treatment BM, the number of clonal cells might be expected to have a proportional relationship to the number of progenitor cells/cancer stem cells. A BM aspirate would contain a number of clonal cells that may numerically reflect this generative population. When the tumor is treated, this representative population is disproportionately lost but drug-resistant cancer stem cells persist, such that the aggregate VDJ% no longer reflects the size of the progenitor compartment. The generative compartment escapes therapy and after therapy can replenish the tumor mass. Morphologically defined BMPC on the other hand, explicitly quantify only PC. The difference between VDJ% and BMPC as measures of clonality may reflect a population(s) of cells that are enumerated by RPCR but remain undetected by morphologic inspection. These cells may be responsible for the correlation between EFS and VDJ% as well as the lack of correlation between EFS and BMPC. Furthermore, these non-PC clonal cells may contribute to malignant clonal expansion.

**Figure 5:**
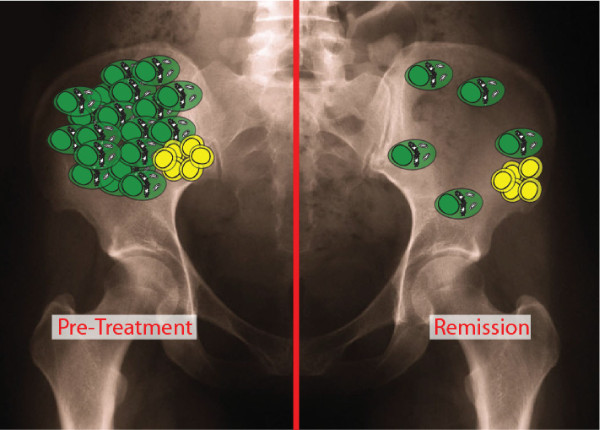
**Hypothesis: a possible perspective on myeloma growth.** Green cells represent plasma cells; yellow cells represent cancer stem cells/myeloma progenitors. This model proposes that the number of plasma cells accurately reflects the number of generative cancer stem cells prior to therapy, after diagnosis or in relapse (left panel) but does not bear a numerical relationship to cancer stem cells in remission (right panel). We speculate that this may be due to selective killing of plasma cells by therapy that spares cancer stem cells. In this context, the pre-treatment VDJ% may correlate with EFS because it is an indirect measure of cancer stem cell activity.

While the VDJ% in pre-treated BMs shows a significant correlation to EFS, BMPC or Mpr levels (as surrogate indicators of PC but not of earlier stage clonotypic cells) do not. We speculate that this may indicate a significant population(s) of cells not detected by routine clinical tests that has a strong influence on the course of disease. Our current methods do not allow us to directly evaluate the nature or number of such non-PC clonotypic cells, but our previous work suggests that they are frequent [2,6,15]. Furthermore, for the patients studied, this cell population appears to be depleted by subsequent ASCT but not by other forms of treatment. One would expect that if there were an even distribution of cell types killed by the therapy, then the tumor burden (VDJ%) in a remission BM would be predictive. Given that this may not always apply, it seems likely that therapy might sometimes have a non-random impact by killing some members of the MM clone but not others, because only some compartments respond. Speculatively, bone marrow prior to treatment (Figure 5, left) contains a population of cells that proportionately represent the number of putative cancer stem cells [4,6]. On the right, a remission bone, plasma cells have been depleted by non-transplant therapy, but progenitors remain, so the sampling no longer predicts the number of cancer stem cells. In this situation, unlike other treatments, ASCT may deplete the progenitor population such that the pre-treatment numbers no longer have predictive value.

## Conclusions

Our work can be interpreted in terms of a clonotypic cancer stem cell population in MM BM [4]. Taken together, our results suggest that except for patients treated with ASCT, tumor burden/cancer stem cell output before treatment is an indirect measure that may reflect the number of MM cancer stem cells and hence event free survival.

## Material and methods

### Patients, clinical data and outcome

BM aspirates were collected from 171 MM patients and the clonotypic IgH VDJ rearrangement was identified in all of them. Written informed consent was obtained, as approved by the University of Alberta and Alberta Health Services Human Ethics Review Committees. One hundred and thirty nine BM aspirate samples (Table 1) were utilized in the pre-treatment study of clonal (VDJ) percent. These included 89 previously untreated MM patients and 50 relapsed/refractory MM patients. BM plasmacytosis (BMPC) and monoclonal protein levels were determined during routine clinical testing and the values used for analysis were obtained from patient records. The % BMPC was determined by the clinical laboratory from Wright-Giemsa stained BM particle crush slides. Thirty-two remission BM aspirates were also analyzed. These samples were drawn from patients at varying time points during remission (e.g. after ASCT or at CR). Patients received initial or new rounds of therapy after the BM aspirate was taken. Patient treatments include single agent or multi agent therapy using combinations of vincristine, doxorubicin, melphalan, prednisone, thalidomide, dexamethasone, cyclophosphamide, lenalidomide or bortezomib and some received an ASCT. Patient samples were unfractionated dry cell pellets stored at −80°C, from which DNA was purified. Remission duration, as defined by event-free survival (EFS), was defined as the number of days between the BM sampling (at diagnosis or relapse) and clinical relapse (defined by a rise in peripheral or urine M-protein level ^40^) or death. Time to progression (TTP) was measured as the number of days between the remission BM sampling and relapse/death. Overall survival (OS) is defined as the number of days from diagnosis until last follow-up or death.

### Cell purification and DNA isolation

BM aspirates were processed to obtain mononuclear cells (BMMC) using Ficoll-Paque PLUS (GE Healthcare, Piscataway, NJ, USA) as described previously [15]. BM mononuclear cells (BMMC) were archived at −80°C as pellets for later DNA isolation; because the entire pellet is used for the DNA extraction, there is no loss of cellular material from the thawed pellet. DNAzol Reagent (Invitrogen, Carlsbad, CA, USA) was used to extract the DNA according to the manufacturer’s instructions. The DNA quantification was performed using the NanoDrop ND-1000 spectrophotometer (NanoDrop Technologies, Wilmington, DE, USA) to ensure equal loading for the PCR applications.

### Patient specific PCR primers

Primers were specific to each patient’s VDJ rearrangement as previously described [40]. Briefly, a panel of 12 V region 5’ primers are used with either an IgA or IgG constant region 3’ primer to PCR amplify any rearranged IgH transcripts, followed by direct DNA sequencing. Primers were designed over the CDR1 or CDR2 region and the CDR3 region. The primers were tested on the unfractionated BMMC sample from the same patient. In parallel with a standard PCR control (water), samples from other patients with similar VH regions controlled for primer specificity. When possible, primers were further validated using single sorted CD38^+^CD138^+^ PCs from the BMMC of the same patient. Twenty-four single PCs were tested by PCR using patient specific primers; a success rate of 75% or higher was required to validate identification of the clonotypic IgH VDJ.

### Clonal cell enumeration by RPCR

RPCR was performed on genomic DNA from BMMCs using the DNA Engine Opticon 2 (Bio-Rad Laboratories, Hercules, CA, USA). The DyNAmo HS SYBR Green qPCR Kit (Finnzymes, Espoo, Finland) was used for the RPCR reaction as follows: 10 μl of DynAmo Master Mix, 0.25μM of each patient specific primer, genomic DNA and water to 20μl. For the patient specific reactions using BMMC samples, 150ng of DNA was added. The control PCR amplified an intron/exon boundary of the β2m gene (to eliminate contamination by cDNA) and 75ng each BM reaction of genomic DNA was added to the PCR to compensate for the two genomic copies of β2m versus the single rearranged clonotypic VDJ copy. The cycling conditions for all the RPCR reactions were 15min at 95°C, 45 cycles of 15sec at 94°C, 30sec at 60°C and 30sec at 72°C, and a final extension phase of 2min at 72°C. Opticon Monitor 3 software (Bio-Rad Laboratories, Hercules, CA, USA) was used to analyze the RPCR data. The threshold was placed at the midway point of the log amplification curve to generate the C(T) value for each RPCR reaction. The amplicon was verified through melting curve analysis.

A cloned amplicon was used to generate a standard curve to determine the exact molecule count for each reaction. The β2m and patient specific IgH VDJ amplicons were cloned using pGEM-T Easy Vector System I (Promega, Madison, WI, USA) and Subcloning Efficiency DH5α cells (Invitrogen, Carlsbad, CA, USA) per manufacturer’s instructions. Plasmids were isolated using QIAprep Spin Miniprep Kit (Qiagen Inc., Mississauga, Ontario, Canada) and the identity of the insert was confirmed by PCR with the primers used to generate the original amplicon. A 10-fold dilution series was constructed and at least three replicates for each dilution were subjected to RPCR analysis concurrent with the BMMC sample. The software generated a standard curve where the slope defines the relationship between the C(T) value to a molecule count. The percentage of MM clonal cells, termed VDJ%, was calculated based on the molecule count of patient specific VDJ targets as compared to β2m the β2M molecule count. Β2M is encoded on chromosome ch15. A subset of MM patients have PC with trisomy 15 and thus likely 3 copies of β2M. This may result in an undefined extent of underestimate for the VDJ% in such patients [41]. However, each MM BM sample analyzed includes a large proportion of normal cells, all of which are diploid. We find that the median % PC in ficoll-purified BMC from MM patients is about 20%, confirming that the majority of BMMC in any given sample are likely to be normal cells with only 2 copies of the B2M gene. Monosomy or trisomy of chromosome 14 would also influence the calculation of VDJ%, depending on which ch14 has been affected; it is likely these would be equivalently distributed in both groups of patients. Given than only one ch14 has the clonotypic IgH VDJ, there is no way to know if the deleted ch14 in monosomies (10% of patients) or the extra ch14 in trisomies (14% of patients) harbors the clonotypic VDJ or an unrearranged/nonproductive VDJ. On a random basis, the affected chromosome will be the productively rearranged VDJ in about half of structural abnormalities, but the prevalence of M protein suggests that for monosomies, the ch14 with a productive (clonotypic) VDJ is not affected. For ch14 translocations, the fact that nearly all such MM make M protein indicates that the productively rearranged VDJ (clonotypic) is not affected by the translocation. Consideration of chromosomal abnormalities as related to RPCR is relevant only if in any given patient all cells have the same chromosomal count, an assumption that is challenged by the presence of normal cells in BMMC and the extensive intra-clonal heterogeneity in individual MM patients [42-45]. These caveats aside, the resulting percentage represents the number of clonal cells present in total BMMC. Where both a pre-treatment and remission VDJ% were available, the relative reduction was calculated by dividing the pre-treatment VDJ% by the remission VDJ%.

Cloned amplicons were generated for only the first 53 patients from the cohort. The C(T) values generated from the individual standard curve reactions were plotted together to form an aggregate regression curve. The resolved slope formula was used to generate new aggregate VDJ% values using the original samples C(T) values where x was the C(T) value and y was the molecule count. A significant correlation between the VDJ% values calculated with the individual standard curves versus those using the aggregate standard curve was found using a Spearman’s correlation (p<0.0001, r=0.5530) and a Fishers Exact test based on the medians (p<0.0001). The VDJ% values calculated using the two methods were plotted in a Kaplan-Meier curve and a logrank test showed that both generate significant associations with EFS. Based on this analysis, for the remaining 118 patients, an aggregate VDJ curve was verified for use in place of a patient specific curve.

To determine sensitivity of the RPCR, DNA from the LP-1 cell line was used. DNA was purified from normal peripheral blood mononuclear cells and spiked with LP-1 DNA. To each VDJ reaction, 150ng of DNA from each dilution was added, and to each β2M reaction, 75ng of DNA was added to compensate for two β2M gene loci compared to the single rearranged VDJ locus.

### Statistical tests

To test for correlations with outcome, including event free survival and overall survival, Kaplan Meier curves were generated using GraphPad Prism 5 and a logrank statistics generated a p-value. Correlation tests were performed using Pearson’s Correlation, Spearman’s Correlation or Fisher Exact tests wherever applicable. Kaplan-Meier curves were generated for data using timed endpoints and longrank tests determined the statistical significance. A Kruskal-Wallis test or an ANOVA was used to compare the medians of more than two groups of data. These tests were done using Sigma Plot Software.

## Abbreviations

ASCT: autologous stem cell transplant; BM: Bone marrow; BMMC: Bone marrow mononuclear cells; BMPC: Bone marrow plasmacytosis; ch: chromosome; CR: Complete remission; EFS: Event free survival; HR: Hazard ratio; IgH: Immunoglobulin heavy chain; ISS: International Staging System; MM: Multiple myeloma; Mpr: Monoclonal protein; OS: Overall survival; PC: Plasma cells; PCR: Polymerase chain reaction; RR: relapsed/refractory; RPCR: Real-time quantitative PCR; TTP: Time to progression; VDJ: Variable, diversity and joining segments of Ig.

## Competing interests

The authors have no conflicts of interest and no competing interests.

## Authors' contributions

KJT designed the experiments, did the work, analyzed the data and wrote the paper. TR collected patient samples, guided the analysis and edited the paper. ARB and LMP directed the work and wrote the paper. All authors read and approved the final manuscript.
